# Extracellular Vesicle-Mediated miR-150-3p Delivery in Joint Homeostasis: A Potential Treatment for Osteoarthritis?

**DOI:** 10.3390/cells11172766

**Published:** 2022-09-05

**Authors:** Huan Wang, Jun Shu, Chengfei Zhang, Yang Wang, Rongxing Shi, Fan Yang, Xuezhang Tang

**Affiliations:** 1 Department of Traditional Chinese Medicine Massage, China-Japan Friendship Hospital, Beijing 100029, China; 2 Institute of Clinical Research, China-Japan Friendship Hospital, Beijing 100029, China; 3 School of Life Sciences, Beijing University of Chinese Medicine, Beijing 100029, China; 4 School of Traditional Chinese Medicine, Beijing University of Chinese Medicine, Beijing 100029, China; 5 Department of Traditional Chinese Medicine Acupuncture, China-Japan Friendship Hospital, Beijing 100029, China

**Keywords:** osteoarthritis, extracellular vesicles, joint homeostasis, innate immune response, miR-150-3p

## Abstract

Background: The disruption of joint homeostasis is a critical event during the process of joint injury in osteoarthritis (OA). As regulatory molecules, microRNAs (miRNAs) can be released from secretory cells and delivered to recipient cells through extracellular vesicles (EVs), thereby playing an important role in regulating joint homeostasis. We hypothesized that the fibroblast-like synoviocytes (FLSs) in healthy joints could release EVs enriched in miRNAs that can maintain joint homeostasis by regulating the signal transduction pathways in the joints, whereby the articular cartilage (AC) is protected from degeneration, and OA progression is delayed. Methods: Via high-throughput sequencing and qPCR, we found that miR-150-3p was enriched in the circulating EVs in healthy rats. Next, we established an in vitro cell model in which chondrocytes were cultured with (i) FLSs transfected with miR-150-3p mimics or (ii) EVs released by FLSs (FLS–EVs) inside the healthy synovial membrane (SM). The transportation mechanism from FLSs to chondrocytes was studied using the EV inhibitor GW4869, and the FLSs were transfected with a miR-150-3p mimic or inhibitor. To assess the therapeutic effect of miR-150-3p-carrying EVs (EVs-150) in vivo, healthy FLS-derived EVs (H-FLS–EVs) were injected into the tail vein of rats with OA at various stages of the pathogenesis and evaluated for the progression of OA. Results: The chondrocytes could uptake fluorescent-labeled miR-150-3p mimics and FLS–EVs, and GW4869 suppressed this uptake. The overexpression of miR-150-3p could significantly reduce the concentrations of pro-inflammatory cytokines in the cell culture medium and the expression of the miR-150-3p target T cell receptor-interacting molecule 14 (Trim14), as well as the innate immune-related factors, including nuclear factor kappa B (NF-κB) and interferon-β (IFN-β). Similarly to the in vitro findings, the miR-150-3p level in the serum EVs was significantly upregulated among the EV-treated rats. In the AC of the OA rat model injected with H-FLS–EVs, the joint degeneration was suppressed, and Type II collagen (COLII) and aggrecan (ACAN) were significantly upregulated, whereas the innate immune-related factors Trim14, NF-κB, and IFN-β were downregulated compared with the levels in the untreated OA rats. Notably, the suppression of joint degeneration was more significant when H-FLS–EVs were administered at the early stages of OA rather than the late stages. Conclusion: H-FLS–EVs protect chondrocyte function and maintain joint homeostasis by modulating the innate immune response by suppressing the Trim14/NF-κB/IFNβ axis. These effects are achieved through the EV-mediated transport of miR-150-3p from the FLSs to the chondrocytes. Our findings show that EV-mediated miR-150-3p can be used to suppress OA, thus providing a novel therapeutic strategy. Additionally, the EV-mediated miR-150-3p transport may also serve as a potential biomarker in the diagnosis, treatment, and prognosis of OA.

## 1. Introduction

Osteoarthritis (OA) is the most common joint disease and has increasingly affected societies worldwide, especially in middle-aged and elderly populations [1,2]. However, current OA treatment strategies, including conservative and surgical treatments, can only serve as the last resort to temporarily relieve the clinical symptoms and joint destruction [3,4,5]. Hence, a new and effective treatment is urgently needed. Furthermore, the onset age of OA has been gradually decreasing, and thus, young people have also increasingly become susceptible to OA [6,7]. However, because of poor attention to early symptoms and the lack of reliable biomarkers for early diagnosis before any irreversible damage occurs, many patients in the early stage of OA often miss the optimal treatment opportunity [8,9]. In fact, timely attention and effective treatment are both important in OA [9,10].

As impaired joint homeostasis is the core of articular cartilage (AC) injury in OA [11], researchers have employed various strategies to maintain joint homeostasis to protect joint health [12,13]. Combined with the definition of “homeostasis”, first proposed by Claude Bernard in 1852 [14], we believe that joint homeostasis refers to the steady state of all joint structures and can be achieved by maintaining the liquid matrix environment surrounding the joints. As the liquid matrix that surrounds the main structures of the joint, synovial fluid is the primary medium for communication between the tissue cells [15,16]. Synoviocytes in the joint can maintain joint homeostasis by releasing bioactive molecules into the synovial fluid, thereby communicating and interacting with the chondrocytes [17,18,19,20]. During the pathogenesis of OA, this homeostasis is impaired. Moreover, as the disease progresses, inflammatory synoviocytes release bioactive substances to anatomical spaces other than the joint cavity, and thus, these substances can disseminate through blood circulation. Consequently, the homeostasis is impaired not only at the injured joint but also at other locations in the body. Therefore, timely intervention and control of the pathological processes underlying the impaired joint homeostasis induced by synovial initiation is a new therapeutic strategy against OA. However, the mechanisms affecting joint homeostasis are currently unclear. To elucidate the mechanism of bioactive substance delivery in joint homeostasis, we established an in vitro cell model in this study and examined the fibroblast-like synoviocytes (FLSs), the chondrocytes, and their surrounding liquid environment as the major study objects. We hypothesized that a mechanism of bioactive substance delivery from FLSs to chondrocytes could impact joint homeostasis in OA.

MicroRNAs (miRNAs) are endogenous, non-coding, single-stranded small RNAs that degrade or block the translation of their target mRNAs and thereby downregulate corresponding proteins [21]. From cell-fate decision to signal transduction, miRNAs are widely involved in cellular processes, including the pathogenesis of OA [22]. In recent years, several miRNAs have been used as biomarkers for the diagnosis or prognosis of several diseases [23,24]. The miRNA-mediated novel therapeutic approaches have been applied to various diseases [21,25]. In this study, we first identified that miRNA-150-3p (miR-150-3p) is downregulated in circulating extracellular vesicles (EVs) with OA, and we then investigated the intercellular delivery mechanism and the biological functions of this miRNA.

Hence, we urgently needed a miRNA “enricher” to enable us to detect critical miRNAs and a miRNA “transporter” to deliver the miRNAs during the communication among different cells [26]. EVs, nanomolecular vesicle-like structures made of a double-layer lipid membrane, can be actively released into the extracellular environment and body fluids by almost all types of cells under physiological and pathological conditions. EVs can transport numerous miRNAs and biologically active substances released by secretory cells to nearby or distant recipient cells. These nanostructures act as specific vectors for information transmission between cells and thereby regulate the functions of the recipient cells [27,28]. We hypothesized that miRNA-containing EVs secreted by FLSs could serve as extracellular regulators of joint homeostasis and thereby regulate the OA-related pathways by targeting chondrocytes as recipient cells.

The innate immune system is the decisive barrier of the body against infections and is involved in the synovial inflammation and AC catabolic events underlying OA pathogenesis [29,30,31]. The T cell receptor (TCR)-interacting molecule (TRIM) family proteins are closely associated with the innate immune response. We found that, as the target gene of miR-150-3p, Trim14 can regulate the expression of the proteins involved in the nuclear factor kappa B (NF-κB) signal transduction pathway, activate the innate immune response, and modulate joint homeostasis in OA. We also demonstrated the effect of a healthy synovial membrane (SM) on joint homeostasis in OA. We investigated the mechanism of the EV-mediated specific delivery of miR-150-3p from FLSs to chondrocytes, the primary effector cells in the synovium and cartilage that participate in joint homeostasis [32,33]. We also investigated the mechanism of joint homeostasis through the involvement of the Trim14/NF-κB/interferon-β (IFN-β) axis in the innate immune response. We preliminarily identified the optimal window for applying miR-150-3p-carrying EVs (EVs-150) as a potential treatment for cartilage protection and OA intervention. These observations provide new insights into whether EV-mediated miRNAs can be used as diagnostic, therapeutic, and prognostic biomarkers in OA, thereby achieving joint protection.

## 2. Methods

### 2.1. Construction and Validation of the Animal Model 

**Figure 1:**
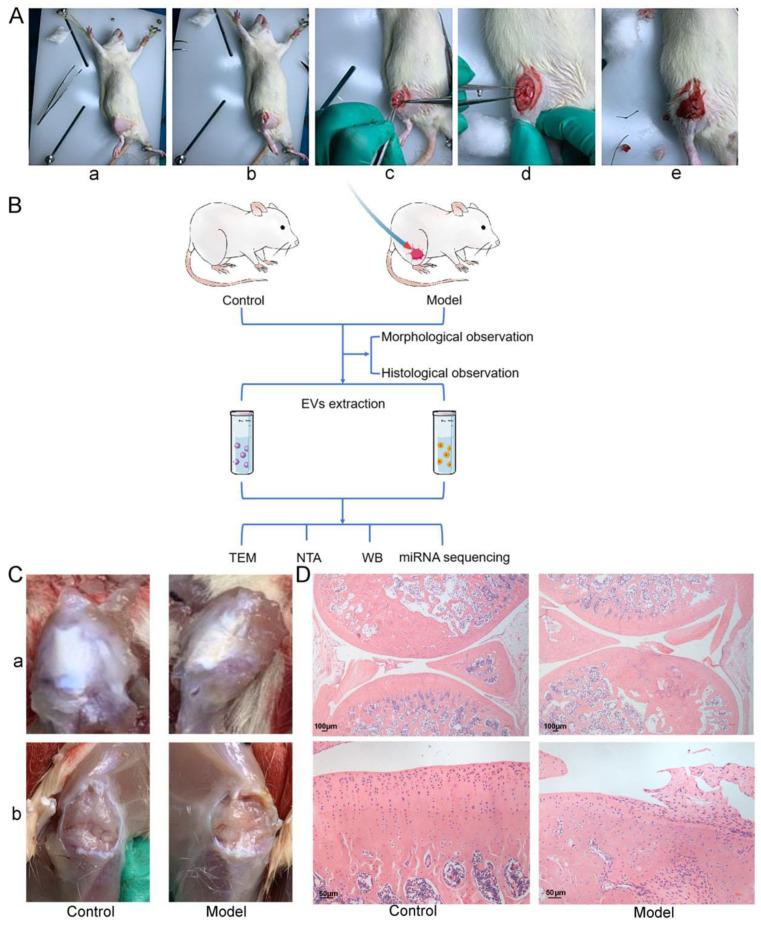
Construction and validation of a rat model of knee OA. (**A**) Establishment of OA model in rats by ALCT method. (**a**) The hair around the knee was removed to expose the surgical incision site. (**b**) After disinfection, the skin and various subcutaneous tissues were sequentially incised using a scalpel. (**c**) The knee joint capsule was exposed laterally. (**d**) The anterior cruciate ligament was cut. (**e**) The muscles, fascia, and skin were sequentially sutured. (**B**) The screening flow for the differentially available miRNAs between the circulating EVs of the control (healthy rats) and the model (OA rats) groups. The circulating EVs were extracted from serum using differential centrifugation, and TEM, NTA, and WB were used to identify the EVs. Then, miRNA sequencing was performed on these EVs. (**C**) Morphological observation. After the skin and subcutaneous tissues surrounding the knee, the knee joints were removed layer by layer. (**a**) The skin on the knee surface was opened. (**b**) Then, the joint cavity was opened, and the SM was exposed. (**D**) Histological observation. The entire knee joints were stained using HE.

Specific-pathogen-free female Wistar rats (8 weeks old and 180–220 g) (SLAC, Shanghai, China) were raised for one week under conventional housing conditions to adapt to the environment before the experiments. After being anesthetized via intraperitoneal injection of 2% pentobarbital sodium (Sinopharm, Beijing, China), the rats were fixed in a supine position. Unilateral knee OA was induced within the rats (model group) through anterior cruciate-ligament transection (ALCT) (Figure 1A). Then, the rats were intramuscularly administered 30,000 U/d penicillin (North China Pharmaceutical Co, Hebei, China) once a day for three consecutive days after the surgery to prevent infection. The mobility of the rats on the surgical side was maintained, and the food and water intake, changes in hair color, incision healing, as well as suture intactness, and incidence of infection were closely monitored. Healthy rats that were not subjected to the surgery were set as the control group and housed under the same conditions. All the animals were provided with water and the conventional pellet food and housed in a quiet and comfortable environment. The study was approved by the Animal Experiment Ethics Committee of China-Japan Friendship Hospital (No. 180117).

Whole blood was collected from the abdominal aorta of the rats in the two groups after 10 weeks and centrifuged at 1900× *g* for 10 min to separate the serum. These serum samples were then stored at −80 °C until needed. The entire knee joint of the rats in each group was harvested, fixed in 4% paraformaldehyde, and stained with hematoxylin and eosin (HE) to validate the successful establishment of the OA animal model.

### 2.2. EV Extraction and Identification

The EVs were isolated from the serum samples from each group by using differential centrifugation as follows: 500× *g* for 10 min, 2000× *g* for 30 min, and 10,000× *g* for 30 min. Afterwards, the supernatants containing the EVs were collected, filtered through 0.22 µm sterile filters into centrifuge tubes, topped up to 20 mL with sterile PBS, and then centrifuged at 120,000× *g* for 1 h (Optima XE, Beckman, Fort Collins, CO, USA). All of the above procedures were conducted at 4 °C in sterile centrifuge tubes. Finally, after carefully removing the supernatant, the pellets were resuspended with sterile PBS and stored at −80 °C for subsequent experiments.

The extracted EVs were identified using transmission electron microscopy (TEM, Tecnai f20, Philips, Amsterdam, Netherlands), nanoparticle tracking analysis (NTA, NS300, NanoSight, Malvern, UK), and Western blotting (WB). After the extracted particles were stained with phosphotungstic-acid solution, TEM was used to observe and photograph the extracted particles and determine whether their morphology was consistent with the general characteristics of EVs. NTA was used to measure the diameter and concentration of the extracted particles (diluted for 30 folds in sterile phosphate-buffered saline [PBS, Corning Incorporated, Corning, NY, USA]). WB was used to detect EV marker proteins, including CD9, CD63, and HSP70 (Abcam, Cambridge, MA, USA), and the detailed specific procedures are illustrated in the “WB” section.

### 2.3. miRNA Sequencing and Validation

The EV miRNAs were analyzed using high-throughput sequencing. The procedures of the bioinformatic analysis were as follows: The raw data were processed and statistically analyzed; then, quality control, length-distribution analysis, database comparison and filtration, gene comparison, and analysis of the gene-expression level were conducted on known and predicted miRNAs to identify the differentially expressed (DE) miRNAs (YanzaiBio Co, Shanghai, China). The DE miRNAs were then distributed on a volcano plot, and a heatmap was employed to cluster these miRNAs according to their expression levels. The DE miRNAs were validated using reverse transcription-quantitative polymerase chain reaction (qPCR) and subjected to gene ontology (GO) and Kyoto Encyclopedia of Genes and Genomes (KEGG) pathway enrichment analyses to predict their target genes, associated pathways, and putative functional roles.

### 2.4. Construction of the In Vitro Cell Model 

#### 2.4.1. Cell Isolation and Culture

The OA rat model was constructed using the methods described in the sub-section “Construction and validation of the animal model”, which should be performed under sterile conditions for the subsequent cell culture. The SM was harvested from the knee joint of the surgical side and rinsed several times with Dulbecco’s PBS (DPBS, Corning Incorporated, Corning, NY, USA). After the removal of the attached surrounding tissues (fat, ligaments, fascia, etc.), the synovium was minced and digested with 5 mL type II collagenase (Sigma-Aldrich, St. Louis, MO, USA) for 4 h at 37 °C. The solution was gently shaken several times during the digestion. The collected cell suspension was filtered through a 100-mesh filter and centrifuged at 300× *g* for 5 min. Then, the cells were seeded into culture flasks and passaged when the cell density reached 70–80%. The cells passaged 3–5 times were used in the experiments. The isolation and culture of the healthy synoviocytes and chondrocytes were performed using the same methods as were used with the OA synoviocytes, except that the chondrocytes were harvested from the AC and then digested for ≥6 h. The morphology of the isolated and cultured synoviocytes and chondrocytes was examined under a light microscope (IX-70, Olympus, Tokyo, Japan).

Flow cytometry (FCM) was utilized to detect the viability of the synoviocytes and chondrocytes, along with the synoviocyte surface markers CD90 (FLSs), CD68 (macrophages), and CD3 (T cells), and the mesenchymal stem cell (MSC) surface markers CD29 and CD105 were also labeled. Vimentin, the characteristic labeled protein of FLSs, was tagged with the SM and FLSs through immunohistochemical (IHC) and immunofluorescence (IF) analysis to further identify FLSs. Toluidine-blue staining was used to examine the developing glycosaminoglycans in the extracellular matrix of the isolated chondrocytes, and their expression of type II collagen (COLII) was assessed via IF analysis. The production of glycosaminoglycans and COLII is characteristic of chondrocytes.

#### 2.4.2. Model Construction

To study the mechanisms of EV-mediated miRNA delivery and its role in OA, we constructed an in vitro OA model. Two different cell types were co-cultured without contacting each other using a transwell system (8 µm, Corning Incorporated, Corning, NY, USA). In this cell model, FLSs and chondrocytes were cultured in the upper and lower chambers as secretory and recipient cells, respectively, with Dulbecco’s modified Eagle’s medium/F12 (DMEM/F12, Gibco, Carlsbad, CA, USA), containing 10% fetal bovine serum (FBS, Gibco, Carlsbad, CA, USA). Bioactive factors can realize the communication between the two cell types through this liquid environment and across the permeable polycarbonate membrane of the transwell system and exert their biological functions.

### 2.5. miRNA Transportation in the In Vitro OA Model 

miR-150-3p mimics were labeled with Cy3 to observe their location. FLSs were transfected with the Cy3-labeled mimics (GenerayBiotech, Shanghai, China) according to the manufacturer’s instructions. The sequences were as follows: miR-150-3p mimic, forward 5′-CUGGUACAGGCCUGGGGGA-3′, reverse 5′-CCCCAGGCCUGUACCAGUU-3′.

The transfected FLSs were seeded in the upper chamber and co-cultured with chondrocytes pre-seeded in the lower chamber. To assess whether the miR-150-3p mimics transfected into the FLSs in the upper chamber had been released and then absorbed by the chondrocytes in the lower chamber, the localization of the red Cy3 fluorescence was characterized. As a control, another group of FLSs was treated with Cy3 that was not conjugated to miR-150-3p and then co-cultured with the chondrocytes. Under a fluorescence microscope, the chondrocytes were examined for the presence of the red Cy3 fluorescence signal to address the effect of Cy3 on them alone.

In addition, to assess whether EVs are involved in the delivery of miR-150-3p from FLSs to chondrocytes, we treated another set of the in vitro OA model transfected with the Cy3-labeled miR-150-3p mimics with the EV inhibitor GW4869 (Sigma-Aldrich, St. Louis, MO, USA) (10 µM). 

### 2.6. FLS–EV Extraction and Identification

Given the high levels of miR-150-3p in the circulating EVs of healthy rats, we considered that miR-150-3p might positively impact the injured joints in OA. However, to determine whether EVs-150 in the circulation are associated with FLSs, which participate in OA progression, and whether FLSs can secrete EVs, we isolated FLSs from healthy rats and cultured them as described above. The FLSs were passaged until they reached the required cell quantity. Then, they were collected, rinsed three times with PBS, and transferred to a fresh serum-free EV medium (Gibco, Carlsbad, CA, USA). After 48 h, the culture supernatant was collected and its EV content was extracted via differential centrifugation as described above. TEM, NTA, and WB (CD9, TSG101, and HSP70 [Abcam, Cambridge, MA, USA]) were used to characterize the EVs.

### 2.7. EV-Uptake Assay

To detect the uptake of FLS–EVs by chondrocytes in vitro, we labeled the FLS–EVs using the PKH67 green-fluorescent kit (Sigma-Aldrich, St. Louis, MO, USA). For subsequent experiments, the EVs were re-extracted via differential centrifugation and quantitated using a bicinchoninic acid (BCA, Sigma-Aldrich, St. Louis, MO, USA) assay. To observe the uptake of EVs by chondrocytes, the chondrocyte nuclei were first labeled with the Hoechst stain (Sigma-Aldrich, St. Louis, MO, USA) for 15 min to aid the localization of the cells, and then, the cells were rinsed three times with PBS. With the addition of the EVs (50 mg/L final concentration), the EV uptake of the chondrocytes was observed and recorded after 1, 6, 12, and 24 h. The other group was pre-treated with GW4869 for 24 h before the addition of the EVs, and the change in fluorescence intensity after the EV blockage was observed.

### 2.8. In Vitro EV-150 Functional Assays 

To further study the functions of EVs, we treated the chondrocytes with GW4869 or FLS–EVs isolated from healthy rats and compared these cells with untreated chondrocytes. qPCR was used to evaluate the effect of the EV treatment on the expression of miR-150-3p in the chondrocytes, to confirm that this miRNA was enriched in EVs and transported by them and to determine whether GW4869 terminated this process.

To assess the regulatory effect of EVs-150 on the chondrocytes of OA, the expression levels of COLII and aggrecan (ACAN) were measured using qPCR and WB. The concentrations of the pro-inflammatory cytokines interleukin-1β (IL-1β), IL-6, and tumor necrosis factor-α (TNF-α) in the chondrocyte culture medium were measured using an enzyme-linked immunosorbent assay (ELISA).

Furthermore, to investigate the mechanism whereby FLS–EVs from healthy rats regulate OA, we predicted the target genes of miR-150-3p and associated pathways via GO enrichment analysis. Given the role of the innate immune response in OA that we showed in our previous study [34], the effects of EVs-150 on the expression of the target gene Trim14 and the downstream genes NF-κB and IFN-β, three genes involved in the innate immune response, were analyzed using qPCR and WB.

To further study the functions of EVs-150, we used TargetScan to predict the targets of miR-150-3p. Consequently, we predicted Trim14 as a potential miR-150-3p target and identified the putative binding site of miR-150-3p on Trim14. These results were validated using the dual luciferase assay. To this end, we constructed a set of pGL3 luciferase reporter vectors with the promoter region composed of the identified miR-150-3p binding sites on the Trim14 promoter. Next, 293T cells (Cell Bank of Chinese Academy of Sciences, Shanghai, China) were transfected with a vector expressing miR-150-3p or the negative control (NC) vector. The sequences (Generay Biotech, Shanghai, China) used for the dual-luciferase assay were: pGL3-Trim14-3′ UTR-WT, TTCTGAGCTGGGGTTTGTGCTGGC; pGL3-Trim14-3′ UTR-MUT, TAGTGAGGTGCCGTTACAGGTGCC; miR-150-3p mimic NC, forward 5′-UUCUCCGAACGUGUCACGUTT-3′, reverse 5′-ACGUGACACGUUCGGAGAATT-3′.

### 2.9. Regulation of the Innate Immune Signaling by miR-150-3p

To elucidate the molecular mechanism underlying the effect of miR-150-3p on joint homeostasis during OA, we transfected FLSs with a miR-150-3p mimic, miR-150-3p inhibitor, or NC by using Lipofectamine 2000 (Invitrogen, Carlsbad, CA, USA) according to the manufacturer’s instructions. Then, the cells were transferred to the upper chamber of the transwell system in the cell model and evaluated for their ability to influence the chondrocytes in the lower chamber. The pro-inflammatory cytokines IL-1β, IL-6, and TNF-α in the culture medium were quantitated using ELISA. The expression levels of COLII, ACAN, Trim14, NF-κB, and IFN-β in the chondrocytes of each group were determined via qPCR and WB. The sequences of the oligonucleotides (Generay Biotech, Shanghai, China) used were as follows: miR-150-3p inhibitor, 5′-UCCCCCAGGCCUGUACCAG-3′; miR-150-3p inhibitor NC, 5′-GGCCUCACCGGGUGUAAAUCAG-3′.

### 2.10. In Vivo EV-150 Functional Assays

Healthy FLS-derived EVs (H-FLS–EVs) were labeled with the DiR fluorescent dye (KeyGEN, Nanjing, China). The labeled EVs were re-extracted to remove impurities and then injected into rats with OA via the caudal vein (the construction of the OA rat model is described above). The fluorescence distribution in the animals was observed after 1, 6, and 24 h via live imaging (IVIS Lumina XRMS Series III, PerkinElmer, Waltham, MA, USA). Likewise, the DiR fluorescent dye without EVs was injected into another set of healthy or OA rats, as a control.

The H-FLS–EVs were injected inside the rats through the tail vein, and four-week treatment in the early stages (early EV-treated group) of OA was compared with the same treatment period in the late stages (late EV-treated group). The rats were grouped and processed as follows: In the early EV-treated group, EV injection (500 µg/kg) was conducted on days 1 and 4, from the fourth to the seventh week after the surgery; in the late EV-treated group, EV injection was conducted on days 1 and 4, from the seventh to the tenth week after the surgery; in the model group, the animals were not subjected to any intervention after the surgery; and in the control group, healthy rats from the same batch of animals that had been used to construct the OA model were used. The behavior of the rats within each group was assessed based on the modified Lequesne MG index [35]. It had the stimulus–response of local pain, altered gait, joint motion range, and joint swelling degree. Blood and joint fluid were collected from each group on the last day of week 10, and the concentrations of the pro-inflammatory cytokines IL-1β, IL-6, and TNF-α were measured using ELISA. The cartilage tissues were harvested and stained with Masson to perform histological grading through the Wakitani scoring system. qPCR was used to measure the miR-150-3p level in the serum EVs of each group. The effects of EVs-150 on the expression levels of COLII, ACAN, Trim14, NF-κB, and IFN-β in the AC of each group were determined via qPCR, WB, and IF to investigate the regulatory functions of EVs-150 in OA. 

### 2.11. Cell Viability and FLS Characterization of FLSs through FCM

The FLSs or chondrocytes were digested with 0.25% trypsin containing 0.05% EDTA (Gibco), centrifuged at 300× *g* for 5 min, collected, and then washed twice in the stain buffer (BD Pharmingen, San Diego, CA, USA). After the cell pellets were resuspended in the stain buffer, the cell concentration in each sample was adjusted to 1 × 10^7^ cells/mL. Then, 100 μL of each cell suspension (approximately 1 × 10^6^ cells) was transferred into an FCM tube. Subsequently, Annexin V-FITC/PI, FITC-labeled anti-CD3 antibody, PE-labeled anti-CD29 antibody (eBioscience, San Diego, CA, USA), PE-labeled anti-CD90 antibody, PE-labeled anti-CD68 antibody, and FITC-labeled anti-CD105 antibody (Abcam, Cambridge, MA, USA) were added into each sample. The samples were then incubated for 20 min at room temperature (20 ± 5 °C) in the dark. The isotype control was likewise prepared using the isotype antibody (BD Pharmingen) at a volume equal to the total volume of the anti-CD antibodies. After the incubation, the samples were mixed with 2 mL stain buffer and centrifuged at 300× *g* for 5 min. The supernatant was discarded, and each cell pellet was resuspended in 0.5 mL stain buffer. FCM analysis (LSRII, BD Biosciences, San Jose, CA, USA) was promptly performed, and approximately 10,000 cells per sample were analyzed. The collected data were exported in the FCS3.0 format and analyzed using the Flowjo software (Tree Star Inc., Ashland, OR, USA). The proportion and intensity of the antigen expression were determined based on the isotype controls. 

### 2.12. Histological Analysis

The entire knee joint or femoral condyle with complete AC was harvested, fixed in 4% paraformaldehyde (Sigma-Aldrich, St. Louis, MO, USA) for 24 h, and then decalcified using the EDTA (Servicebio Tech., Wuhan, China) method for 28 d with daily replacement of the decalcifying solution. After dehydration by an ethanol gradient, vitrification by xylene, and paraffin embedding, the trimmed wax block was cut into 4 μm-thick sections using a microtome (RM2016, Leica, Wetzlar, Germany). The tissue sections were then dewaxed and rehydrated using conventional procedures for subsequent use.

The entire joints from the control and model groups were stained with HE (Servicebio Tech., Wuhan, China). The morphological characteristics of the tissues were observed under a microscope to validate the successful construction of the OA model. AC morphology and collagen maturity were assessed via Masson’s staining (Sigma-Aldrich, St. Louis, MO, USA) to evaluate the OA progression after the EV-150 treatment.

### 2.13. Toluidine Blue Staining of Chondrocytes

Chondrocyte suspension was collected, and 3.5 × 10^4^ cells were transferred into each well of a 24-well plate containing sterile coverslips. After overnight standing, the culture supernatant was removed, and the cells were incubated in 4% paraformaldehyde for 20 min at room temperature. Afterwards, the cells were washed with PBS and stained with toluidine blue (Solarbio, Beijing, China) for 30 min. Finally, they were rinsed with anhydrous ethanol and observed and photographed under a microscope.

### 2.14. IHC/IF Analysis

#### 2.14.1. IHC Analysis of SM

The SM tissue required for separated FLSs was labeled Vimentin to determine the purity of FLSs in SM. After the SM sections, antigen repair was undergone and then incubation with the anti-Vimentin primary antibody (Abcam, Cambridge, MA, USA), and goat anti-rabbit HRP secondary antibody (Abcam, Cambridge, MA, USA) was introduced for the final detection. The nuclei were counterstained using hematoxylin (Servicebio Tech., Wuhan, China) and sealed. The positive FLS expression was observed using an inverted fluorescence microscope.

#### 2.14.2. IF Analysis of FLSs and Chondrocytes

FLSs or chondrocytes grown on coverslips were fixed in 4% paraformaldehyde, permeabilized and blocked using conventional procedures, and then incubated with the anti-Vimentin or anti-COLII primary antibody (Abcam, Cambridge, MA, USA). Goat anti-rabbit IgG-555 fluorescent secondary antibody (Invitrogen, Carlsbad, CA, USA) was employed for the detection, and the samples were mounted with a mounting agent containing DAPI (Burlingame, CA, USA) to stain the nuclei.

#### 2.14.3. IF Analysis of AC

To evaluate the therapeutic potential of EVs-150 in OA, IF colocalization analysis was performed to observe the extent of the AC injury and repair and the changes in the related signaling pathways. After antigen retrieval, tissue sections were incubated with the anti-COLII, anti-ACAN, anti–NF-κB (all from Abcam, Cambridge, MA, USA), and anti-Trim14 (Novus Biologicals, Littleton, CO, USA) antibodies as the primary antibodies and then with the Cy3- or Alexa fluor-488-labeled secondary antibody (Abcam, Cambridge, MA, USA) for fluorescent detection. The cell nucleus was stained with DAPI. The tissue sections were immediately examined and photographed under an inverted fluorescent microscope (IX-73, Olympus, Tokyo, Japan) to determine the fluorescence intensity and localization.

### 2.15. WB Analysis

The cells or tissues were lysed, and the total protein concentration was measured using the BCA assay. Equal quantities of proteins were resolved using SDS-PAGE and then transferred onto a PVDF membrane (Merck Millipore, Billerica, MA, USA). Next, the membrane was blocked at 37 °C for 2 h and then incubated with the primary antibody (anti-COLII, anti-ACAN, anti-NF-κB, anti-IFN-β, anti-GAPDH [all from Abcam, Cambridge, MA, USA], or anti-Trim14 [Novus Biologicals] antibody) at 4 °C overnight. The membrane was washed three times with PBST (PBS with Tween 20, Solarbio) and then incubated with the horseradish-peroxidase-labeled anti-mouse or anti-rabbit IgG secondary antibody (Jackson Immunoresearch, West Grove, PA, USA) (diluted 1: 10,000 with PBST) at 37 °C for 2 h. Finally, the membrane was washed five times with PBST and developed using chemiluminescence (BeyoECL Star [Beyotime, Shanghai, China]). The results were analyzed using the ImageJ software (NIH, Bethesda, MA, USA). 

### 2.16. qPCR

Total RNA was extracted, and cDNA was synthesized using the RNeasy Mini Kit (QIAGEN, Duesseldorf, Germany), PrimeScript RT Master Mix, PrimeScript II 1st Strand cDNA Synthesis Kit (TaKaRa, Tokyo, Japan), and Power SYBR Green PCR Master Mix (Thermo Fisher Scientific, Waltham, MA, USA) according to the instructions of the manufacturers. The relative expression levels of miR-150-3p, COLII, ACAN, Trim14, NF-κB, and IFN-β were measured via qPCR using the ΔΔCt method. For normalization, U6 and cel-miR39 were used as the internal and external small RNA controls (for miR-150-3p), and GADPH was used as the internal control for the mRNAs. The results were normalized, and the specificity of the amplification was determined based on the presence of a single peak in the melting curve. The sequences of the primers (Sangon Biotech, Shanghai, China) used were as follows: miR-150-3p, stem-loop 5′-GTCGTATCCAGTGCAGGGTCCGAGGTATTCGCACTGGATACGACTCCCCC-3′, forward 5′-GCGCCTGGTACAGGCCT-3′; universal downstream primer for stem-loop method, 5′-GTGCAGGGTCCGAGGT-3′; U6, reverse transcription 5′-CGCTTCACGAATTTGCGTGTCAT-3′, forward 5′-GCTTCGGCAGCACATATACTAAAAT-3′, reverse 5′-CGCTTCACGAATTTGCGTGTCAT-3′; cel-miR-39, stem-loop 5′-GTCGTATCCAGTGCAGGGTCCGAGGTATTCGCACTGGATACGACCAAGCT-3′, forward 5′-GGCCTCACCGGGTGTAAATCAG-3′; COLII, forward 5′-ACTTAACATCCAAGGCCGCT-3′, reverse 5′-ACAATATTTGCCTCAGTTTGTGC-3′; ACAN, forward 5′-AGAGGCAGAGGGACTTTCGGT-3′, reverse 5′-AGAGGCAGAGGGACTTTCGGT-3′; Trim14, forward 5′-GTGAATACTTACAGTGCCTTGC-3′, reverse 5′-GACCCAGACCAGAACCCT-3′; NF-κB, forward 5′-GCACGGATGACAGAGGCGTGTATAAGG-3′, reverse 5′-GGCGGATGATCTCCTTCTCTCTGTCTG-3′; IFN-β, forward 5′-ATTGCGTTCCTGCTGTGC-3′, reverse 5′-GTCCGAATGCTAGTGCTTTGTC-3′; GAPDH, forward 5′-AGACAGCCGCATCTTCTTGT-3′, reverse 5′-CTTGCCGTGGGTAGAGTCAT-3′.

### 2.17. Enzyme-Linked Immunosorbent Assay 

The IL-1β, IL-6, and TNF-α levels in cell-culture media, sera, and synovial fluid were measured using ELISA according to the instructions of the manufacturer of the ELISA kit (Abcam, Cambridge, MA, USA).

### 2.18. Statistical Analysis

All the data are expressed as mean ± SEM and statistically processed using the SPSS 22.0 software (SPSS, Inc., Chicago, IL, USA) and GraphPad Prism 9.0 software (GraphPad Software, Inc., La Jolla, CA, USA). Comparison between the two groups and among more groups was performed using the Student’s *t*-test and one-way ANOVA, respectively. *p* < 0.05 was considered to indicate statistical significance. 

## 3. Results

### 3.1. Discovery of the Association of EVs-150 with OA

To identify the miRNAs loaded into EVs at different levels between OA and healthy rats, we first established a rat model of knee OA (described in the Materials and Methods section). After 10 weeks, we analyzed the knee joints of the rats in the control and model groups using HE staining. We found that the synovial tissues in the control group were smooth and shiny. In contrast, those in the model group were significantly thickened and had a rough texture and yellow appearance (Figure 1C). The results from the HE staining of the joints further validated that the animal model was successfully established. The rats in the control group had a healthy, continuous, and intact synovium that enveloped the tissues and structures within the joint cavity, and the synoviocytes were neatly arranged as a thin layer. However, the rats in the model group had thickened synovial tissues with proliferated, aggregated, and disorganized synoviocytes. Furthermore, the control group had a continuous and intact AC tidal-line layer, and the superficial, transitional, radial, and calcified zones were clear. In contrast, the AC in the model group had an abraded superficial layer and a disorganized, shifted, and thinned tidal-line layer. The cell layer was arranged in a disorderly manner and exhibited calcification (Figure 1D).

**Figure 2:**
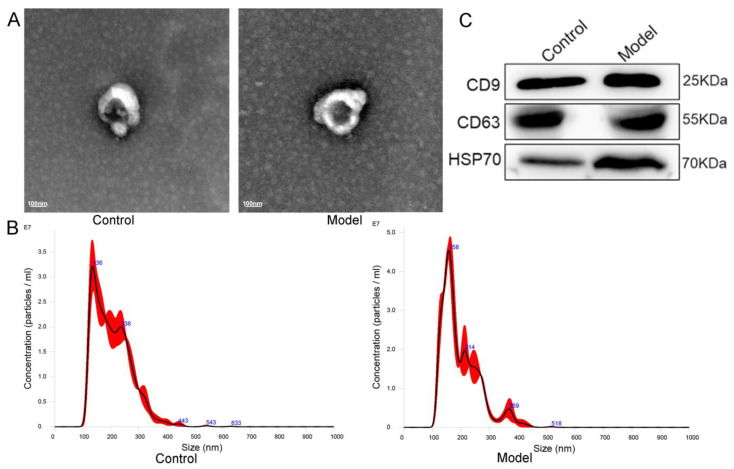
EVs were isolated using differential centrifugation and identified using TEM, NTA, and WB. (**A**) TEM observation of the EV morphology. (**B**) NTA measurement of the EV particle distribution and concentration. (**C**) WB analysis of the EV markers CD9, CD63, and HSP70.

Next, we extracted circulating EVs from serum samples to characterize the miRNA content of these EVs. The characteristics of EVs include morphology, size distribution, quantification, and surface marker expression. TEM, NTA, and WB analysis of the three positive proteins was employed to identify the EVs extracted from the control and the model groups. Under TEM, cup-shaped or round, hollow spherical vesicles were revealed (Figure 2A), and the NTA results showed the sizes and concentrations of the extracted particles (Figure 2B). Moreover, the WB results showed that the extracted EVs were enriched in the EV markers CD9, CD63, and Hsp70 (Figure 2C). These data were consistent with the characteristics of EVs, depicting the successful extraction of circulating EVs.

**Figure 3:**
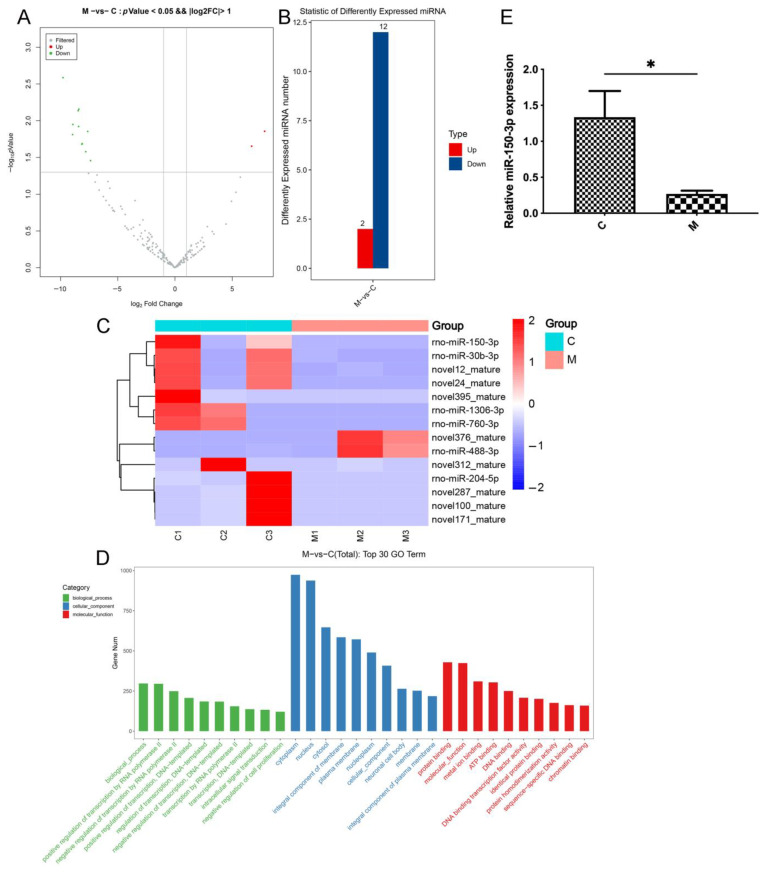
RNA sequencing analyzed DE miRNAs in EVs between the control (C) and model (M) groups. (**A**) Volcano plot of differentially present miRNAs. Gray indicates the miRNAs present at similar levels between the two groups; red indicates the significantly higher presence of miRNAs; and green indicates the significantly lower presence of miRNAs; X-axis is log_2_ Fold Change, and Y-axis is −log_10_ *p*-value. *p* < 0.05, |log_2_ fold change| > 2. (**B**) Fourteen miRNAs were significantly differentially depicted, including twelve upregulated and two downregulated miRNAs. (**C**) Heatmap of the DE miRNAs. Red and blue indicate high and low levels, respectively. (**D**) Gene ontology analyses were performed. (**E**) qPCR validation of the miR-150-3p enrichment in the EVs. Three independent experiments indicate the data as mean ± SEM (*n* = 3). * *p* < 0.05.

miRNAs are the main molecules in EVs and are involved in pathways important in joint injury and repair in OA by regulating the protein levels of target mRNAs. To assess the OA-induced changes in the miRNA levels in EVs, we performed deep sequencing of the miRNAs in the circulating EVs from healthy and OA rats (BioProject ID: PRJNA753999). Among the 233 known and 344 predicted miRNAs in all the samples, we identified the 14 miRNAs that were the most significantly differentially represented between the two groups, including 12 upregulated and 2 downregulated miRNAs. GO enrichment analysis was performed to evaluate the functions of the DE miRNAs. The sequencing results showed that miR-150-3p was over-represented in the control (C) group compared with the level in the model (M) group (log_2_ fold change: 8.43) (Figure 3A–D). The qPCR results confirmed that the miR-150-3p level in the circulating EVs was significantly different between the two groups (*p* < 0.05) (Figure 3E). These results indicated that EVs-150 might be associated with the pathogenesis of OA. Thus, in the subsequent experiments, we assessed the role of miR-150-3p in OA.

### 3.2. Construction of the In Vitro OA Cell Model

To clarify the substance delivery by EVs-150 and the regulatory relationship among different cell types in OA, we established an in vitro cell model that simulates OA.

**Figure 4:**
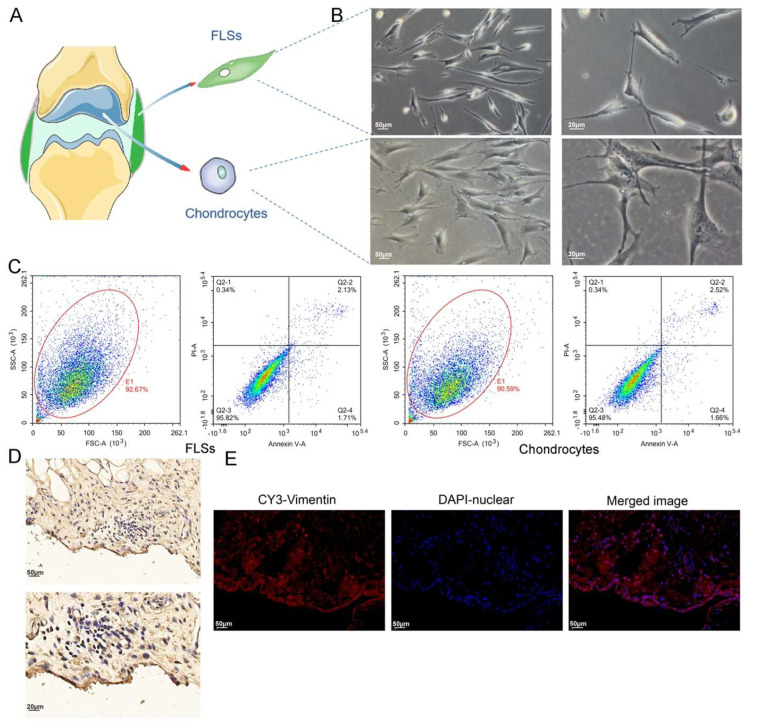
Labeling of FLSs in SM, and cell viability detection for isolated FLSs and chondrocytes. (**A**) FLSs and chondrocytes obtained from SM and AC of rats, respectively. (**B**) Observation of FLSs and chondrocytes using light microscopy. (**C**) FCM was used to determine the viability of FLSs and chondrocytes. (**D**) Vimentin IHC staining of SM. (**E**) Vimentin IF staining of SM.

We isolated FLSs and chondrocytes from the SM and AC tissues of OA rats through collagenase treatment, respectively (Figure 4A). The isolated synoviocytes were cultured, and the cultured cells primarily consisted of FLSs after several passagings. Under an inverted microscope, the FLSs exhibited columnar, spindle-shaped, and occasionally polygonal morphology, with an oval nucleus in the center. The FLSs in the culture formed clusters and grew in a polarized fashion. In contrast, the chondrocytes adhered to the substratum, extended pseudopodia, and exhibited a spindle, star, or polygon shape. Their nucleus was oval or round, clear, and located in the center. The chondrocytes in the culture showed fusion growth and grew in clusters (Figure 4B).

FCM was used to determine the viability of the FLSs and the isolated chondrocytes. The results revealed that the total number of viable cells from the two types of cells was more than 95%. Moreover, the total number of cells during early and late apoptosis or necrosis was less than 5%, which could be utilized for subsequent experiments (Figure 4C).

**Figure 5:**
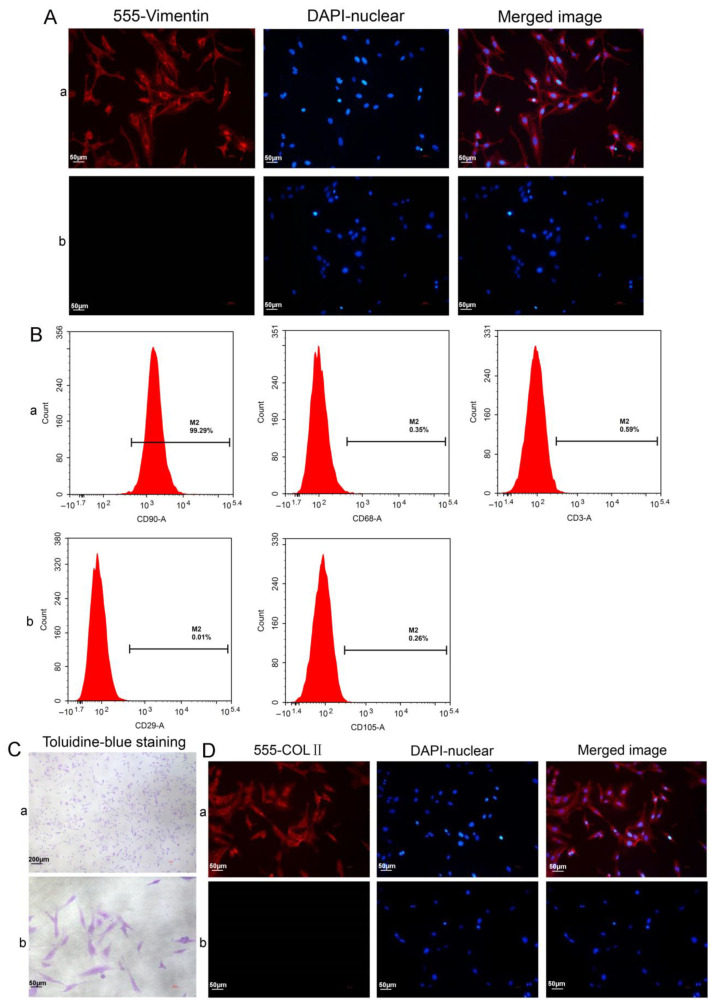
Characterization of the synoviocytes and chondrocytes. (**A**) Vimentin IF staining of FLSs: (**a**) test cells; (**b**) isotype controls. (**B**) The surface markers were analyzed using FCM: (**a**) the synoviocyte surface markers of CD90 (FLSs), CD68 (macrophages), and CD3 (T cells); (**b**) the MSC surface markers of CD29 and CD105. (**C**) Characterization of the chondrocytes using toluidine blue staining. (**D**) Characterization of the chondrocytes with COLII immunofluorescence analysis: (**a**) test cells; (**b**) isotype controls.

To further label FLSs, Vimentin, the characteristic labeling protein of FLSs, was labeled in the SM tissue required for the FLS culture. We observed that the SM in the field of view under the light microscope was all over the brown-yellow positive cells after the IHC staining of the SM (Figure 4D). This was established by the IF staining results of the SM. Moreover, it was observed under the fluorescence microscope that the red Vimentin was mainly located in the cytoplasm, especially in the lining layer of the SM, with a large amount of red fluorescence concentration. It indicated that FLSs were the primary cell type in the SM (Figure 4E), the same as our previous study [34]. The FLSs isolated from the SM were labeled with Vimentin, and the results consistently revealed that most cells were Vimentin-positive (Figure 5A).

FCM was employed to analyze several main cell types in the synoviocytes, namely FLSs (CD90), macrophages (CD68), and T cells (CD3), and the results showed that highly pure FLSs (99.29% of the total cells) could be obtained using our isolation and culture methods (Fig. 5B). In addition, the expression rates of the MSC surface markers CD29 and CD105 were 0.01% and 0.26%, respectively. These were negative, excluding the possibility that our isolated cells from the SM were MCSs. Proteoglycans and COLII are the main components of the cartilage matrix and thus the characteristic markers of chondrocytes. The basic toluidine blue stain combines with the acidic proteoglycans in chondrocytes to present a bluish-purple color (Figure 5C). COLII IF staining showed that the cytoplasm and nuclei of the chondrocytes displayed red and blue fluorescence, respectively. Accordingly, the chondrocytes were COLII-positive. These results demonstrated that the cells we isolated and cultured displayed the characteristics of chondrocytes (Figure 5D).

We established a transwell-based cell model in which FLSs and chondrocytes were co-cultured in the upper and lower chambers in a common liquid environment, respectively, to simulate the interaction between the two major cell types in a joint. The subsequent experiments were performed using this in vitro OA model.

### 3.3. Pathways of Intercellular miRNA Transport 

Various cell types actively secrete miRNAs entering the blood circulation. They can be delivered into recipient cells by vectors, such as EVs, thereby affecting the functions of the recipient cells. Healthy synoviocytes, together with the synovial fluid they secret, play important roles in the protection of chondrocytes against injury. We determined whether FLSs, the largest cell population in synoviocytes, can secret extracellular miRNAs transported to chondrocytes. 

FLSs transfected with Cy3-labeled miR-150-3p mimics were transferred to the upper chamber of a transwell to be co-cultured with chondrocytes plated in the lower chamber. The presence of the red Cy3 fluorescence in the chondrocytes suggested that the Cy3-labeled miR-150-3p mimics were transferred from the FLSs in the upper chamber to the chondrocytes in the lower chamber. FLSs can secrete extracellular miRNAs that can be absorbed by chondrocytes as the recipient cells, suggesting miRNA-mediated signaling from FLSs to chondrocytes.

**Figure 6:**
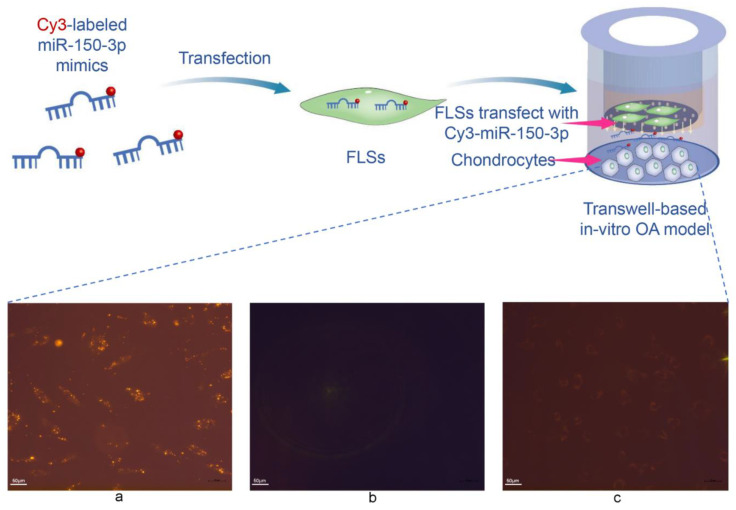
The ability of exosomes to transport miRNAs. miRNA transport in the transwell-based in vitro OA model. (**a**) FLSs were transfected with a miR-150-3p mimic labeled with Cy3 (red fluorescence) and co-cultured with chondrocytes. (**b**) FLSs were treated with Cy3 alone. (**c**) GW4869 inhibition of EV formation.

We considered that FLS–EVs might carry the miRNAs to be transported to the chondrocytes and thus treated the transwell co-culture system with GW4869, an inhibitor of EV secretion. The GW4869 treatment reduced the intensity of the red fluorescence in the chondrocytes in the lower chamber, indicating that inhibiting the secretion of EVs decreased the delivery of miR-150-3p to the chondrocytes. Thus, FLS–EVs may serve as carriers for the miRNAs to be transported from FLSs to chondrocytes (Figure 6).

### 3.4. Uptake of FLS–EVs by Chondrocytes

**Figure 7:**
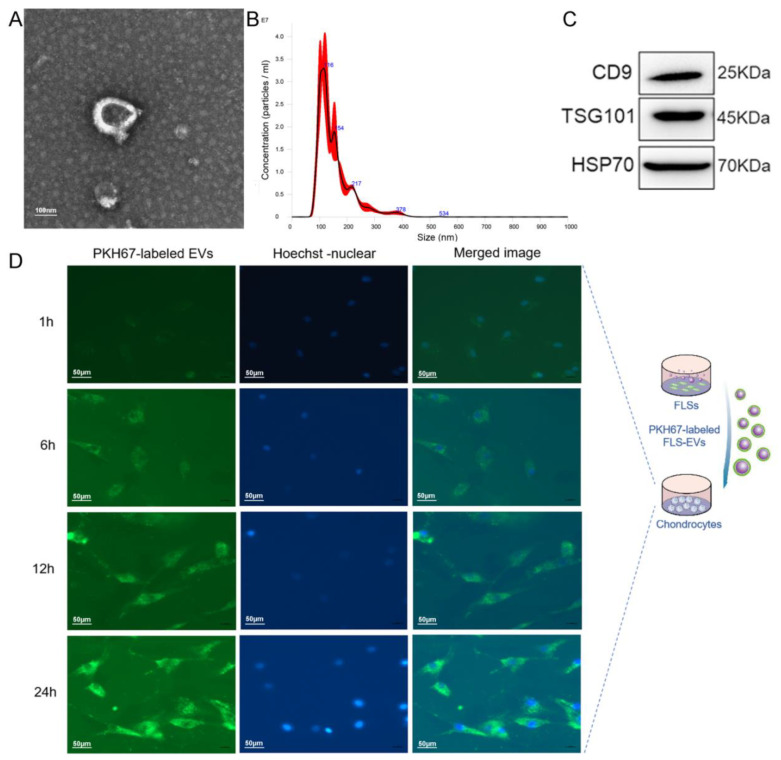
The identification FLS–EVs and uptake of FLS–EVs by chondrocytes. (**A**) TEM observation of the EV morphology. (**B**) NTA measuring of the EV particle distribution and concentration. (**C**) WB analysis of the EV markers CD9, TSG101, and HSP70. (**D**) Representative immunofluorescence images of chondrocytes at various time points, showing the uptake of PKH67 (green)-labeled EVs. Hoechst (blue) staining was performed to label the nuclei.

EVs are secreted by cells containing miRNAs and are the primary mechanism of intercellular miRNA transport. To determine whether FLSs can secrete EVs, we cultured FLSs isolated from the SM of healthy rats, extracted the EVs in the cell-culture supernatant via differential centrifugation, and then characterized them. These EVs were presented as hollow spherical vesicles under TEM (Figure 7A). In addition, NTA revealed that the main peak for the particle size corresponded to 116 nm (Figure 7B), and the particles were positive for the EV-specific protein markers CD9, TSG101, and HSP70, indicating that FLSs can secrete EVs (Figure 7C).

As the transwell assay can only prove that substance transport did occur between the cells in the upper and lower chambers, we conducted an EV-tracking experiment to assess whether chondrocytes could uptake EVs secreted by FLSs. The FLS–EVs were labeled with the fluorescent dye PKH67 and then co-cultured with chondrocytes. After 6 h, green fluorescence was observed in the perinuclear region of the chondrocytes, demonstrating that the PKH67-labeled EVs had been endocytosed by the chondrocytes. The intensity of the green fluorescence in the chondrocytes gradually increased with time, especially in the perinuclear region, and reached saturation at 24 h, indicating the high absorption of the FLS–EVs by the chondrocytes (Figure 7D).

Our results indicated that FLSs could secrete EVs that effectively transport miR-150-3p to chondrocytes.

### 3.5. EVs-150 Promote Repair of the Chondrocytes in the In Vitro OA Cell Model

Considering that H-FLS–EVs may have a positive impact on the maintenance of joint homeostasis, we co-cultured FLS–EVs isolated from the SM of healthy rats with OA chondrocytes for 24 h. The qPCR results showed that the miR-150-3p level in the chondrocytes was significantly increased compared with the control group (*p* < 0.05), further confirming that EVs deliver miR-150-3p from FLSs to chondrocytes in vitro. In contrast, the GW4869 treatment suppressed the increase in miR-150-3p level in the chondrocytes.

**Figure 8:**
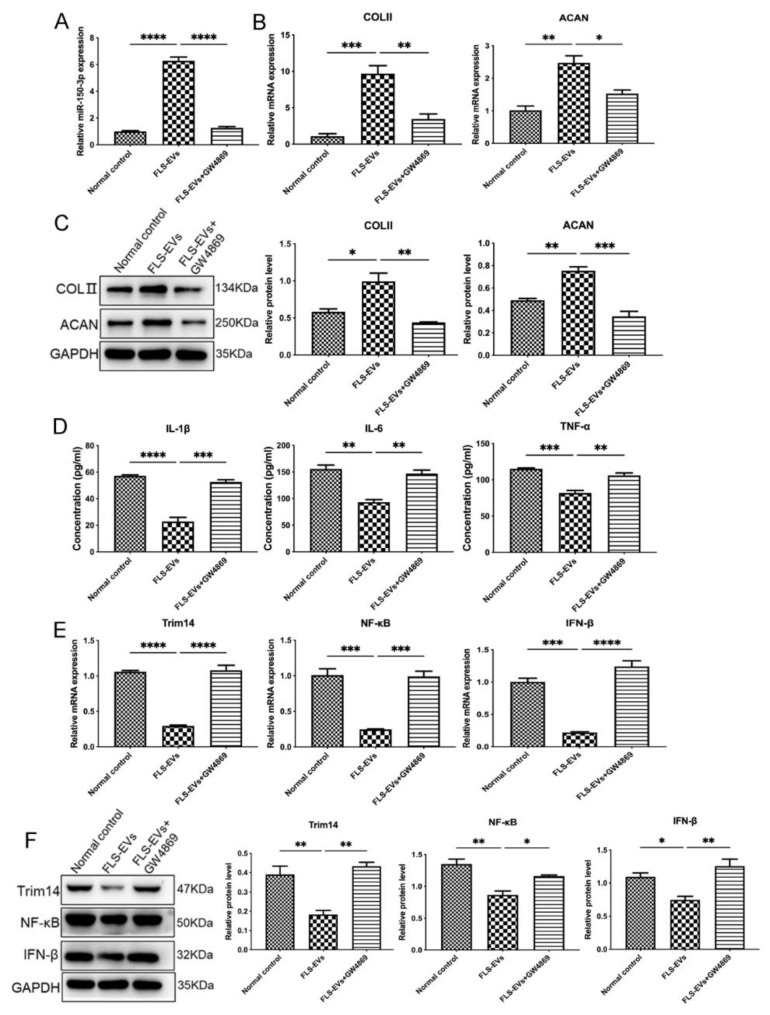
The mechanism underlying the effect of EVs-150 on chondrocyte repair. (**A**) qPCR analysis of the miR-150-3p expression in chondrocytes treated with H-FLS–EVs or GW4869. (**B**) qPCR analysis of the COLII and ACAN mRNA levels in chondrocytes upon H-FLS–EV treatment. (**C**) The COLII and ACAN protein levels in chondrocytes using WB upon H-FLS–EV treatment. (**D**) ELISA-based quantitation of the pro-inflammatory cytokines IL-1β, IL-6, and TNF-α in the chondrocyte culture medium. (**E**) qPCR analysis of Trim14, NF-κB, and IFN-β mRNA levels. (**F**) WB analysis of Trim14, NF-κB, and IFN-β protein levels. The data are expressed as mean ± SEM from three independent experiments. **** *p* < 0.00001, *** *p* < 0.0001, ** *p* < 0.01, * *p* < 0.05.

Moreover, the COLII and ACAN expression in the chondrocytes treated with the H-FLS–EVs was enhanced, and the concentrations of the pro-inflammatory cytokines IL-1β, IL-6, and TNF-α in the chondrocyte culture medium were decreased, demonstrating that EVs-150 may promote chondrocyte repair through the regulation of the expression levels of the target genes and related pathways. GO and KEGG enrichment analyses of the target genes and pathways revealed that miR-150-3p could target the Trim14 and NF-κB pathways of the innate immune response. Adding H-FLS–EVs decreased the mRNA and protein levels of Trim14 and its downstream genes NF-κB and IFN-β. However, the addition of GW4869 suppressed this effect, indicating that EVs-150 regulated Trim14, NF-κB, and IFN-β expression levels at the transcript level (Figure 8).

### 3.6. miR-150-3p Regulates the Innate Immune Signal Transduction to Maintain Joint Homeostasis

**Figure 9:**
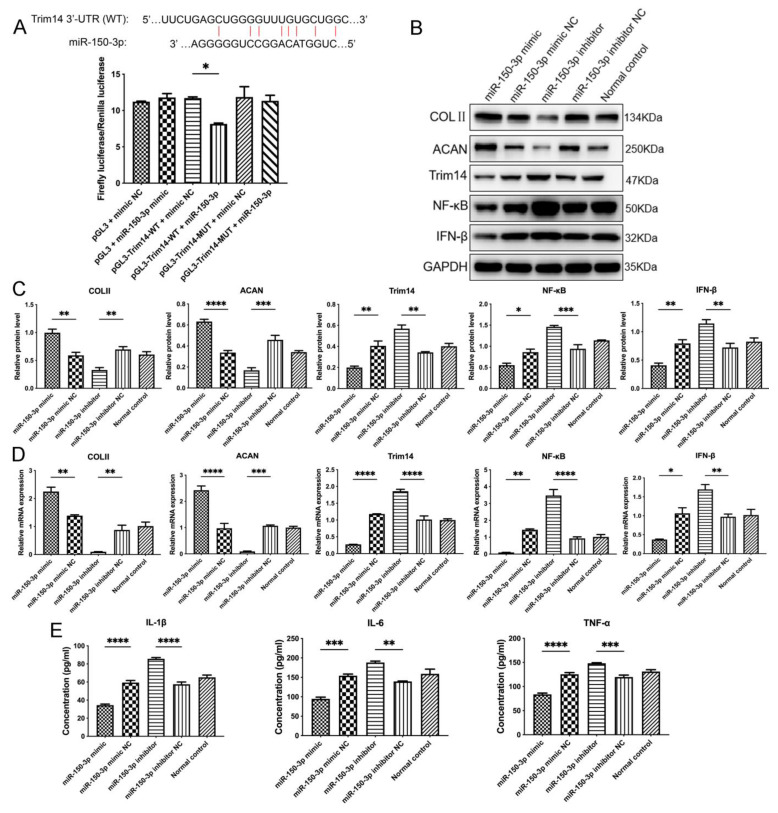
Validation of the miR-150-3p target and the regulatory effect of miR-150-3p on the innate immune signaling and joint homeostasis. (**A**) A miR-150-3p binding site on Trim14 and validation of miR-150-3p targeting Trim14 through the dual luciferase assay. (**B**,**C**) COLII, ACAN, Trim14, NF-κB, and IFN-β protein levels were analyzed using WB. (**D**) qPCR analysis of the Trim14, NF-κB, IFN-β, COLII, and ACAN mRNA levels in chondrocytes after miR-150-3p interference. (**E**) ELISA-based quantitation of the pro-inflammatory cytokines IL-1β, IL-6, and TNF-α in the chondrocyte culture medium. The data are expressed as mean ± SEM from three independent experiments. **** *p* < 0.00001, *** *p* < 0.0001, ** *p* < 0.01, * *p* < 0.05.

To elucidate the mechanism underlying the regulation of Trim14 expression by miR-150-3p and its relationship with the NF-κB signaling pathway, we analyzed the binding site between miR-150-3p and the 3′ end of Trim14 by using TargetScan. A plausible binding site was identified in the promoter region of Trim14. As shown in Figure 9A, the promoter fragments were significantly inhibited by miR-150-3p expression, indicating that Trim14 is a direct target of miR-150-3p.

To further investigate the mechanisms of the chondrocyte repair and maintenance of joint homeostasis induced by H-FLS–EVs, we examined the effect of miR-150-3p on the Trim14/NF-κB/IFN-β signal transduction in the OA-related innate immune response. FLSs transfected with plasmids overexpressing miR-150-3p mimics were co-cultured with OA chondrocytes in the in vitro model. The COLII, ACAN, Trim14, NF-κB, and IFN-β expression levels in the chondrocytes were measured using WB and qPCR. The miR-150-3p mimics significantly upregulated the COLII and ACAN protein levels but downregulated the Trim14, NF-κB, and IFN-β protein levels in the chondrocytes (Figure 9B,C). The corresponding mRNA levels had a similar trend with the protein levels. Furthermore, the miR-150-3p mimics also significantly decreased the release of the pro-inflammatory cytokines IL-1β, IL-6, and TNF-α into the culture medium.

We also assessed whether the miR-150-3p inhibitor had the opposite effect. It was found that the inhibitor enhanced the release of the pro-inflammatory cytokines, reduced the COLII and ACAN expression in the chondrocytes, and activated the Trim14/NF-κB/IFN-β pathway. These observations demonstrated that miR-150-3p could effectively regulate the Trim14/NF-κB/IFN-β axis of the innate immune response and stimulate FLSs to influence chondrocytes and the co-culture microenvironment, indicating that miR-150-3p may be a potential therapeutic target in OA and other joint diseases (Figure 9).

### 3.7. H-FLS–EVs Promote Joint Protection in OA

**Figure 10:**
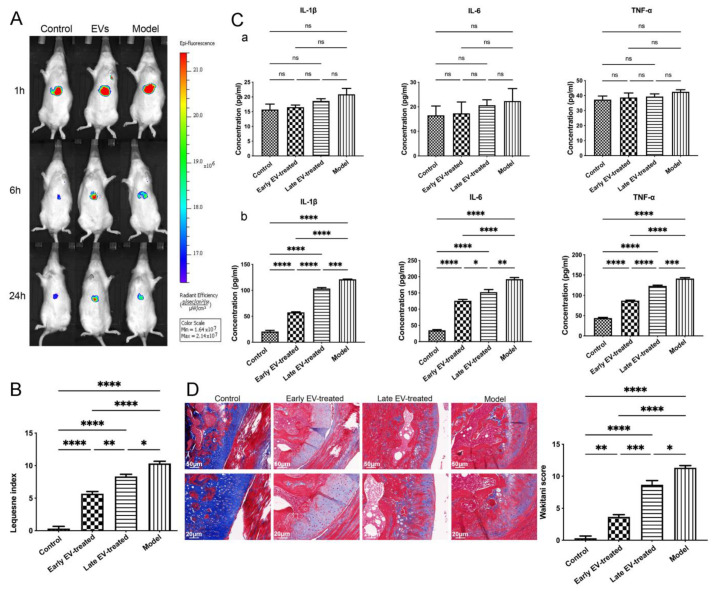
In vivo tracking and role of H-FLS–EVs in the joint homeostasis. (**A**) In vivo EV tracking using live imaging. (**B**) Lequesne index of rats’ behavior. (**C**) ELISA-based quantitation of the pro-inflammatory cytokines IL-1β, IL-6, and TNF-α in serum (**a**) and synovial fluid (**b**). (**D**) Histological analysis of EV-treated AC using Masson’s staining. The data are expressed as mean ± SEM from three independent experiments. **** *p* < 0.00001, *** *p* < 0.0001, ** *p* < 0.01, * *p* < 0.05; ns, not significant.

The miR-150-3p carried by H-FLS–EVs has an inhibitory effect on the Trim14/NF-κB/IFN-β signal transduction during the innate immune response in vitro. We evaluated the effects of this miRNA on joint homeostasis in OA in vivo. Fluorophore-labeled H-FLS–EVs were injected into the rats via the tail vein and tracked in vivo. Most fluorophores clustered around the liver in each group and gradually dimmed and disappeared by 24 h, in line with the metabolism process of medicines in rats. Any signal in other regions was assessed until the fluorophores disappeared from the imaging system with time quenching. This result is inconsistent with previous studies in the literature, which reported evident fluorophore distribution lasting several weeks in the injured joints [36]. Our results indicated a strong liver expression, and the small level of fluorescence that may be present at the joints was not enough to be detected. We deduced that H-FLS–EVs injected through the caudal vein could exert physiological effects such as circulating EVs-miRNA through systemic circulation. This is also one of the reasons why we selected blood as the most accessible sample to extract EVs for miRNA sequencing at the beginning of the study (Figure 10A).

Despite the loss of the evidence of intraarticular fluorescence, we have demonstrated the joint protection effect of FLS–EVs in vivo by other means, similar to the ones described in vitro. The joints of the rats treated with H-FLS–EVs (early EV-treated group and late EV-treated group) were compared with the joints of the rats in the model group (rats with OA) and the control group (healthy rats) without any EV intervention. We first measured the concentrations of inflammatory mediators in the synovial fluid, and the results showed lower concentrations in the early EV-treated and late EV-treated groups than in the model group, especially in the early EV-treated group, indicating an improved liquid-matrix environment in the joint. Before sample collection, various indices of the rats in each group were also consistent with the above trend, according to a Lequesne MG index, suggesting the improvement effect of H-FLS–EVs on behavior (Figure 10B). However, no significant differences were observed in the serum levels of the inflammatory mediators among the groups. Moreover, no abnormal level of circulating inflammatory mediator was detected in comparing the model group with the control group (Figure 10C). This result indicated that OA tends to cause sterile, low-grade joint inflammation. Even though the inflammatory joint in OA could deliver some substances to influence disease progression via blood circulation, OA is not a systemic inflammatory disease, based on the traditional definition. The widely recognized inflammatory mediators in the blood cannot be considered indicators for a sensitive diagnosis and prognosis in OA. However, the level of miR-150-3p in the serum EVs was also significantly upregulated in the EV-treated rats, similar to the findings in vitro. This result again emphasizes the importance of our studies on EVs-150.

**Figure 11:**
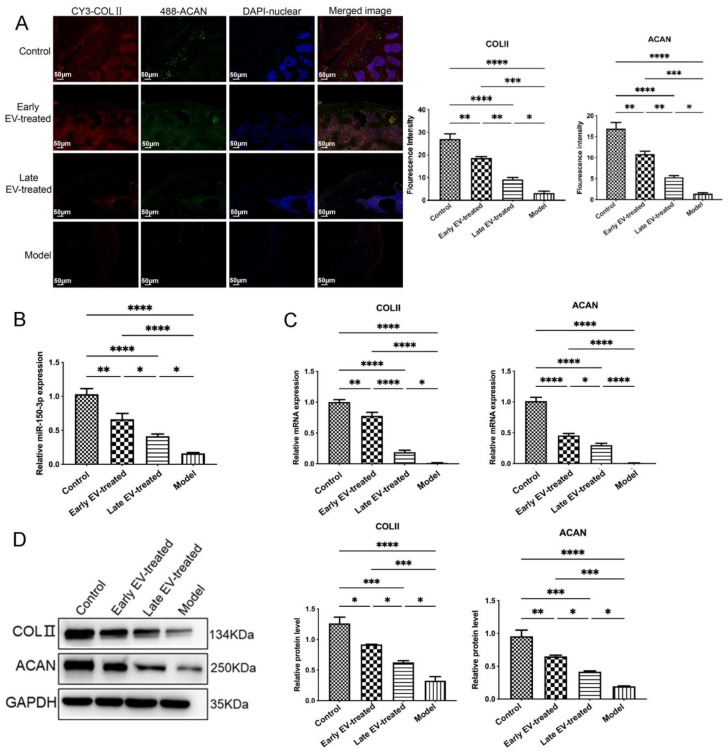
Regulatory effect of EVs-150 on AC protection in vivo. (**A**) Observation of the levels and localization of COLII and ACAN in the AC using IF staining. (**B**) qPCR analysis of the miR-150-3p expression in the serum EVs after EV treatment. (**C**) qPCR analysis of the COLII and ACAN mRNA levels in the AC after EV treatment. (**D**) The COLII and ACAN protein levels were analyzed in the AC using WB after EV treatment. The data are expressed as mean ± SEM from three independent experiments. **** *p* < 0.00001, *** *p* < 0.0001, ** *p* < 0.01, * *p* < 0.05.

In EV-treated rats, although the levels of some inflammatory markers in the bloodstream were not substantially changed compared with the control levels, the AC showed a clear improvement. We performed the histological and characteristic analysis of the AC in each group. Masson’s staining and IF revealed that, compared with the rats in other groups, the rats in group OA had severe joint wear and loss of the cartilage matrix, disordered chondrocyte arrangement, and significantly reduced the COLII and ACAN expression. Cartilage damage and matrix loss were also observed in the early EV-treated and late EV-treated groups, unlike in the control group. However, these changes were not as severe as those in the model group, which showed thinning of the cartilage matrix and chondrocyte clustering. They decreased the COLII and ACAN levels in the cartilage matrix. The AC injury severity in the early EV-treated group was less than that in the late EV-treated group. It showed a slightly thinner cartilage matrix compared with that in the control group, a lighter degree of decrease in the COLII and ACAN levels in the cartilage matrix, and significantly better AC conditions than those in the model and late EV-treated group (Figure 10D and Figure 11).

Treatment with H-FLS–EVs reduced the inflammation level and AC injury in OA rats. The early FLS–EV intervention significantly suppressed the progression of OA. It effectively prevented the exacerbation of the AC injury caused by the knee instability even more than the late intervention. This observation highlights the importance of starting the treatment as early as possible.

To further evaluate the effect of the EV-150 treatment on the innate immune signal transduction pathway, we measured the levels of miR-150-3p, its target gene Trim14, and the genes involved in the related pathways in the AC of each group. Compared with the levels in healthy rats, in the AC of OA rats the miR-150-3p level was significantly decreased. In contrast, the mRNA and protein levels of Trim14, NF-κB, and IFN-β were significantly increased, suggesting that the innate immune pathway was activated. After the injection of H-FLS–EVs, the abundance of miR-150-3p was partially restored. Concurrently, the miR-150-3p accumulation induced by EVs interrupted the Trim14/NF-κB/IFN-β axis and inhibited joint inflammation.

Co-staining, the AC for Trim 14 and NF-κB, showed the colocalization of these proteins and their upregulation during OA. However, the EV-150 treatment decreased their levels, consistent with the above-mentioned morphological observations on the AC in the different groups. We observed that several target proteins were expressed in the AC at different levels among the different groups. Thus, the levels of Trim14 and NF-κB in the model group were the highest, followed by those in the early EV-treated and the late EV-treated groups, whereas these proteins were barely detected in the joints of healthy rats. Conversely, the levels of COLII and ACAN showed the opposite expression pattern.

**Figure 12:**
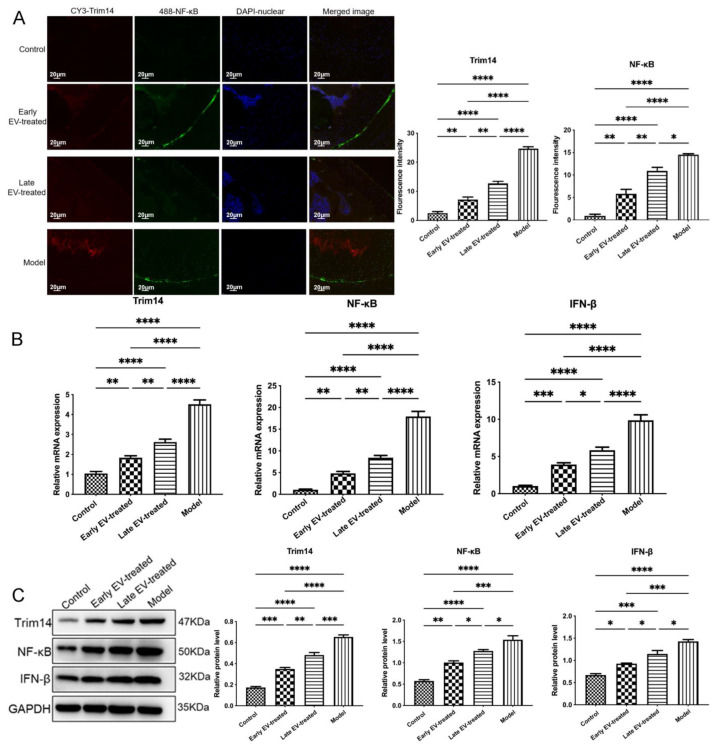
Regulatory effect of EVs-150 on the Trim14/NF-κB/IFN-β axis in vivo. (**A**) The levels and localization of Trim14 and NF-κB in the AC were observed through IF staining. (**B**) qPCR analysis of the Trim14, NF-κB, and IFN-β mRNA levels in the AC after EV treatment. (**C**) After EV treatment, the Trim14, NF-κB, and IFN-β protein levels in the AC were analyzed using WB. The data are expressed as mean ± SEM (*n* = 3) from three independent experiments. **** *p* < 0.00001, *** *p* < 0.0001, ** *p* < 0.01, * *p* < 0.05.

**Figure 13:**
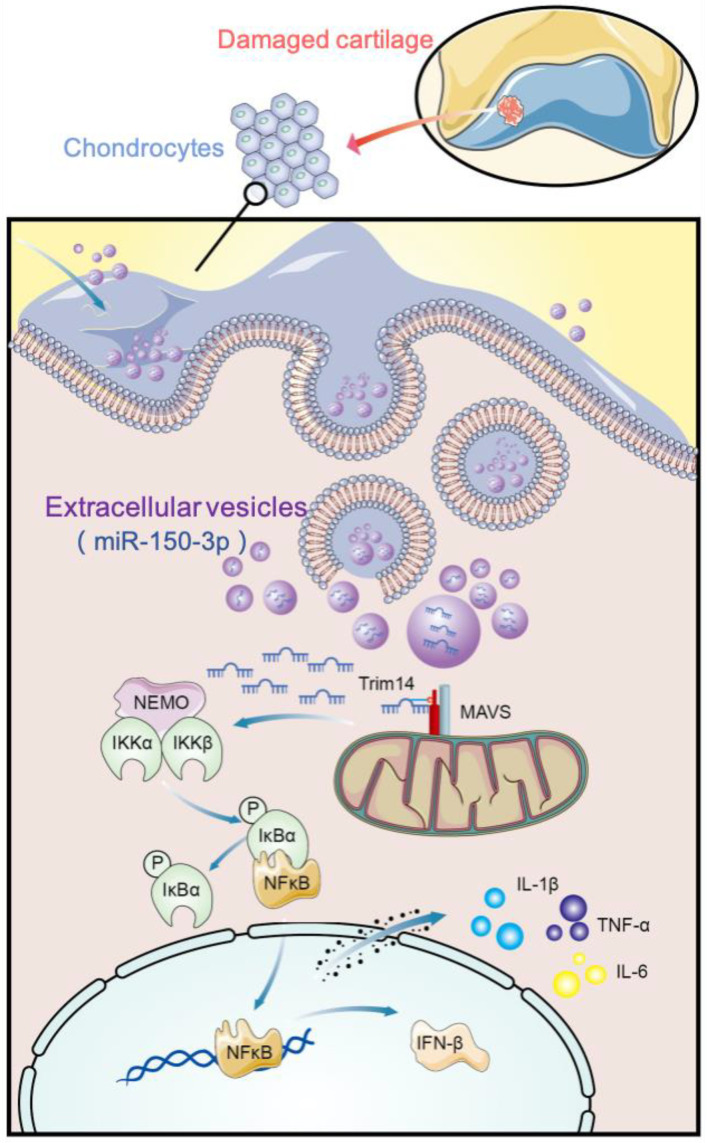
Therapeutic mechanisms of FLS-derived EVs-150. EVs released by the FLSs in the healthy SM were collected, and these EVs could deliver miR-150-3p to chondrocytes. When OA occurs, Trim14 is recruited to the IKK complex by binding to NEMO, a large multi-unit complex that consists of two catalytic subunits (IKKα and IKKβ) and the regulatory subunit IKKγ (which is also known as NEMO, an essential modulator of NF-κB expression). IKK promotes the phosphorylation of IκBα and p65 as well as NF-κB activation and IFN-β expression. After the immune response was activated, the primary pro-inflammatory cytokines IL-1β, IL-6, and TNF-α were expressed, and the various cytokines and chemokines were synthesized and released. Some of these inflammatory mediators were detected in the joint tissues and synovial fluid of OA rats. These mediators can accelerate chondrocyte degradation and metabolism, thereby injuring the joint and causing a series of clinical symptoms. EVs-150 from healthy FLSs could regulate the mRNA level of the target gene Trim14, thereby effectively inhibiting the activation of the positive feedback loop formed by the Trim14/NF-κB/IFN-β axis of the innate immune response, consequently exerting a suppressive effect on the OA processes indicated above.

In addition, Trim14, COLII, and ACAN were found mainly concentrated in the cytoplasm. However, NF-κB was localized in the cytoplasm and nucleus to varying degrees among the different groups. In the control group, NF-κB was at the resting state, localized in the cytoplasm. At this state, NF-κB interacts with its suppressor IκBα to form a non-active complex. When OA occurs, activated IκB kinase (IKK) leads to the phosphorylation of IκBα, and consequently, NF-κB becomes activated and translocates into the nucleus. This process could be suppressed to varying degrees using EV-150 treatment. We also found that the region with the highest fluorescence intensity was concentrated in the wear and ruptured areas of the AC, indicating that they were all involved in joint injury and repair during OA (Figure 11 and Figure 12). The joint homeostasis and repair of the AC injury were stimulated via regulating the innate immune signal transduction pathways, demonstrating that EVs-150 obtained from healthy FLSs show great potential in joint protection. In Figure 13, we have summarized the therapeutic mechanisms of the FLS-derived EVs-150. 

## 4. Discussions

A healthy SM is critical for maintaining joint homeostasis and cartilage integrity [37]. However, the details of the interactions among the tissues in the joints and their specific contribution to the maintenance of joint homeostasis are still unclear. In this study, we demonstrated that H-FLS–EVs could act as vectors for cell-to-cell communication and participate in the maintenance of joint homeostasis through the innate immune response by delivering miR-150-3p to the chondrocytes. Thus, we preliminarily proved the importance of “early intervention”. These results show the therapeutic potentials of H-FLS–EVs in protecting joints and delaying the pathogenesis in OA through miRNA-mediated gene regulation.

As most previous OA studies have focused only on the impact of aberrant joint biomechanics on OA pathology [38], other mechanisms promoting cartilage injury and progressive OA have been neglected. The joint should be viewed as a complex structure to be comprehensively analyzed, a structure wherein the tissue components all take concerted actions to maintain the homeostasis of the internal environment [39]. Hence, in this study, we proposed the concept of “joint homeostasis”. The inflammatory state of the culture medium in direct contact with the two joint-derived cell types in the in vitro model and the synovial fluid in the in vivo model was used as the reference evidence to assess joint homeostasis. Considering the anatomical adjacency and functional interdependence of the AC and SM, alongside the importance of synovial pathology in AC degradation and OA progression, as was demonstrated in our previous study [34], the SM, AC, and the liquid environment between the two were selected as the main subjects to study the mechanism and effect of FLS–EVs on chondrocytes.

miRNAs are regulatory molecules packaged into EVs and delivered from secretory cells to recipient cells to exert functions [23,40,41]. Through bioinformatic analysis, miR-150-3p was depicted at a high level in EVs in the serum of healthy rats. However, it was significantly lower in OA rats, indicating that miR-150-3p could be an indispensable miRNA in joint homeostasis and cartilage protection. 

We hypothesized that FLS–EVs could influence OA progression by acting as extracellular factors that regulate joint homeostasis and AC health. Hence, to further and better simulate the interaction system between the SM and AC and to determine the substance delivery and regulatory relationship between the two, we constructed an in vitro cell model, which mimics the joint cavity and thereby simulates the paracrine mechanism of substances released by FLSs and transported to chondrocytes via the microenvironment. This model also acts as a microscopic circulatory system that simulates the long-distance paracrine mechanism involving chondrocytes as the signaling cells secreting substances that are circulated through the blood to the joints [42].

The past decade has witnessed a rapid increase in the understanding of the sources, characteristics, functions, and pathways of EVs. So far, the studies believe that almost all mammalian cell types, including fibroblasts and dendritic cell lines, MSCs, and dendritic cells, are EV secretory parental cells. MSCs from bone marrow, umbilical cord, and other tissues are the most commonly used EV secretory cells due to their differentiation potential, secretion, immune regulation, and therapeutic functions. They have been widely reported in treating a variety of diseases [43,44]. In recent years, the therapeutic potential of EVs obtained from MSCs in OA has also received increasing attention. Researchers have observed that it can protect OA joints from injury by promoting cartilage repair, suppressing synovial inflammation, and mediating subchondral bone remodeling [28].

Unlike previous studies utilizing mesenchymal stem cells as the primary secretory cells [45,46,47], our study showed that FLSs, the most common cell type in the SM with OA, could also secrete EVs. It is primarily based on the importance of FLSs in maintaining internal joint homeostasis in OA. In the closed joint cavity, chondrocytes depend on various bioactive substances secreted into the synovial fluid using synoviocytes to maintain joint health or alleviate, induce, or exacerbate joint pathology [48,49,50]. FLSs, the primary cell type that constitutes the inner membrane structure of the SM, no longer act as innocent bystanders in the progression of joint disease but actively influence joint inflammation and destruction [51]. FLSs, as an initiation factor, and the synovial fluid, as the mediator generated by them, regulate the transportation of substances among cells and facilitate the primary conditions for the interactions among the various tissues in the environment [50,52]. FLSs and FLS–EVs are closely associated with joint homeostasis and cartilage health, but they have rarely been reported. FLS–EVs are a new attempt to treat OA based on joint anatomy and OA pathogenesis, which is different from previous studies [45,46,47]. In the field of OA, the analysis of miRNAs in FLS–EVs can better understand the pathogenesis of OA and develop new treatment strategies suitable for patients [53]. This study could provide some new ideas and evidence for the further application of FLS–EVs in OA research. However, their transformation and production will be long-term, and in-depth studies are needed to validate the efficacy and optimize the process [54].

These outstanding issues and challenges in developing EV-related technologies have been juxtaposed with the promising development of the material delivery functions of EVs in treating and diagnosing diseases. Based on some studies, not all EVs can carry miRNAs to participate in cell-to-cell communication and thus play a part in biological processes. Moreover, the stoichiometry of miRNAs in EVs and whether EVs are more likely to function through protein or RNA are inconsistent across different studies [55,56,57]. These studies reflect the main reasons for limiting the application of EVs, due to the high heterogeneity of cell types, experimental methods, dose regimens, and even experimental operations used in many studies, leading to the inconsistency of the source, quality, and related technologies of EVs. The latest guidance requirements for EVs (MISEV2018) attempt to solve and unify these problems [58]. However, due to the reasons mentioned above, the recognition of EV function and the effect of EVs have not reached a consensus until now. In addition, it is difficult to monitor and maintain cell viability, potency, and EV transformation during the manufacturing process of EV preparations. Achieving a standardized method for producing and treating a wide variety of EV preparations is impossible.

In the future, newer technologies and scientific methods may apply EVs in OA research. Several studies have confirmed the biological function of EV-mediated miRNAs, the communication effect, and the advantage between cells [19,23]. For instance, Zhou et al. [53] believed that although synovial miRNAs are rapidly degraded, EVs can stabilize these molecules. Thus, EVs enhance their ability to alter the proliferation, differentiation, survival, and inflammatory activity of other cells.

However, previous studies have focused on investigating various pathological states [59,60], and a few have examined the possible roles of bioactive substances obtained from healthy synoviocytes. We proposed the idea of using H-FLS–EVs as a therapeutic measure in OA. However, the study on EV-mediated miRNAs is currently in its initial stage, and whether they can be used for joint protection in OA remains unclear and needs further investigation.

Our findings have clarified the intercellular transport mechanism of miRNAs and demonstrated that EVs can carry miR-150-3p from FLSs to chondrocytes. Healthy FLSs, like mesenchymal stem cells [61], can also play a beneficial role in OA by releasing EVs, and this effect is sufficient to repair the joint and AC significantly. Our study repeatedly emphasized the importance of a good SM condition in maintaining joint health. On this basis, we determined that the EVs-150-mediated intervention of OA at an early stage is more effective than later. Thus, an early diagnosis is crucial to apply this intervention as soon as possible.

EV-mediated miRNAs have sufficient sensitivity and specificity and the potential to serve as simple and accurate biomarkers of OA [62,63]. The changes in the level of EVs-150 are critical for the expression levels of COLII and ACAN and the activities of related pathways, as well as the levels of downstream inflammatory mediators, indicating that the OA treatment strategy is based on EVs-150.

There are few reports on the biological functions of the miR-150-3p-mediated regulation of Trim14 expression. Our study showed that Trim14 is a direct target gene of miR-150-3p, and EVs-150 can influence OA by modulating the innate immune response by regulating Trim14 expression and the NF-κB signal transduction pathway. The NF-κB signaling plays a critical role in the activating of the innate immune response in OA. Many key effectors during OA development and progression are under the control of NF-κB. Many pathways involved in NF-κB activation have been well studied [64,65], but research on the regulation mechanism of Trim14–NF-κB in OA has rarely been reported. Previous studies have reported that Trim14 is a mitochondrial adaptor located on the outer mitochondrial membrane and is involved in the innate immune response to infections and tumors [66,67]. Our study found that EVs-150 can target Trim14 and regulate its expression to affect sterile inflammation, such as OA. We observed that miR-150-3p downregulates Trim14 and thereby reduces the activities of the related NF-κB pathways and that H-FLS–EVs can be used to inhibit the Trim14/NF-κB/IFNβ axis for the treatment of OA.

The rats used in our experiments are highly anatomically suitable for studying various human diseases, including OA and other diseases involving joint injury [68,69]. Rat genes are highly homologous to human genes and are commonly used for OA research [70]. We selected the rat model of OA based on ALCT, a modeling method in common use for OA, which can change the stability of joints and simulate joint wear and degeneration in OA [71].

The crosstalk among cells is essential during OA pathogenesis [72,73,74], and the crosstalk of the Trim14/NF-κB/IFN-β axis and other pathways may also occur in OA. We did not exclude the possibility of substance transportation among other structures in joints. Whether EV-mediated miR-150-3p in the circulation can stimulate the pathogenesis of OA at distal sites, and how it can be used as a diagnostic marker and therapeutic agent in OA, needs in-depth analyses. FLS–EVs from healthy SM can regulate the Trim14/NF-κB/IFN-β axis of the innate immune response by delivering miR-150-3p to chondrocytes to maintain joint homeostasis, repair AC injury, and delay OA progression. The earlier this intervention is provided, the more effective it is in preventing joint degeneration. These important findings may serve as a basis for further studies on the mechanisms of OA and provide an attractive source of biomarker candidates for monitoring joint health during OA. They may also lead to the development of novel approaches for the treatment of OA.

## Data Availability

All data generated or analyzed during this study are included in this published article and are available from the corresponding author upon reasonable request.

## Abbreviations

OA, osteoarthritis; miRNA, microRNA; miR-150-3p, miRNA-150-3p; EV, extracellular vesicle; FLS, fibroblast-like synoviocyte; FLS–EVs, EVs derived from FLSs; healthy FLS-derived EVs, H-FLS–EVs; EVs-150, miR-150-3p-carrying EVs; AC, articular cartilage; SM, synovial membrane; TRIM14, T cell receptor (TCR)-interacting molecule 14; NF-κB, nuclear factor kappa B; IFN-β, interferon-β; COLII, Type II collagen; ACAN, aggrecan; IL-1β, interleukin-1β; IL-6, interleukin-6; TNF-α, tumor necrosis factor-α; IKK, IκB kinase; ALCT, anterior cruciate-ligament transection; C group, control group; M group, model group; NC, negative control; DE, differentially expressed; HE, hematoxylin and eosin; TEM, transmission electron microscopy; NTA, nanoparticle tracking analysis; qPCR, quantitative polymerase chain reaction; WB, Western blotting; GO, gene ontology; KEGG, Kyoto Encyclopedia of Genes and Genomes; BCA, bicinchoninic acid; IF, immunofluorescence; ELISA, enzyme-linked immunosorbent assay; FCM, flow cytometry; PBS, phosphate-buffered saline; DPBS, Dulbecco’s phosphate-buffered saline; DMEM/F12, Dulbecco’s modified Eagle’s medium/F12; FBS, fetal bovine serum.
